# Supplementary figures and images for: Cell behavior on silica-hydroxyapatite coaxial composite
Source: PLoS One. 2021 May 11;16(5):e0246256. doi: 10.1371/journal.pone.0246256 (PMC8112647; doi:10.1371/journal.pone.0246256)

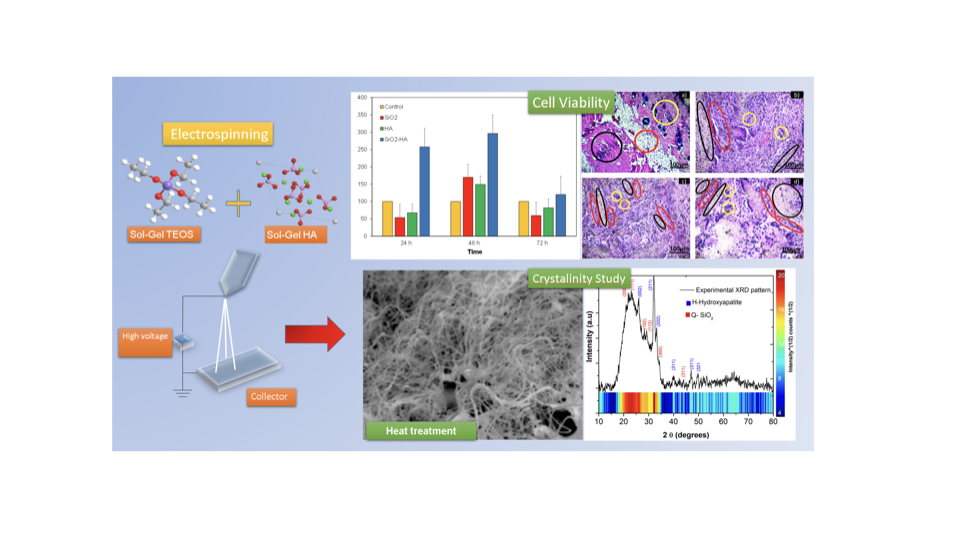

Supplement: S1 Fig — (TIFF) [file pone.0246256.s001.tiff]
