# Supplementary material for: Cell behavior on silica-hydroxyapatite coaxial composite
Source: PLoS One. 2021 May 11;16(5):e0246256. doi: 10.1371/journal.pone.0246256 (PMC8112647; doi:10.1371/journal.pone.0246256)
Supplement: S1 Data — (DOCX) [file pone.0246256.s002.docx]

Table 2. Results obtained from the XRD peak fitting by the Pseudo-Voigt method

| Peak | Peak Center | | Area | | Max Height | | FWHM | | Resolution | | CumArea(0) | |
| --- | --- | --- | --- | --- | --- | --- | --- | --- | --- | --- | --- | --- |
|  | HA | SiO_2_-HA | HA | SiO_2_-HA | HA | SiO_2_-HA | HA | SiO_2_-HA | HA | SiO_2_-HA | HA | SiO_2_-HA |
| 1 | 22.1071 | 22.107 | 0.0675685 | 0.456602 | 0.0921901 | 0.274822 | 0.465161 | 1.05771 | 1.25236 | 0.516723 | 0.0337842 | 0.228301 |
| 2 | 23.142 | 23.065 | 0.0602987 | 0.456347 | 0.0824588 | 0.274663 | 0.464482 | 1.05773 | 3.67154 | 1.56394 | 0.0301493 | 0.228174 |
| 3 | 26.1679 | 25.964 | 0.16245 | 0.583477 | 0.223523 | 0.351185 | 0.462453 | 1.05771 | 2.67063 | 0.999 | 0.0812251 | 0.291738 |
| 4 | 28.687 | 28.301 | 0.1254829 | 0.400134 | 0.0750685 | 0.240829 | 0.236403 | 1.05773 | 0.702736 | 0.592508 | 0.0383192 | 0.200067 |
| 5 | 29.5200 | 29.4 | 0.1480236 | 0.451074 | 0.115676 | 0.271495 | 0.289031 | 1.05771 | 0.668302 | 1.52263 | 0.0168607 | 0.225537 |
| 6 | 32.0178 | 32.222 | 0.456092 | 1.0788 | 0.628668 | 0.649317 | 0.462035 | 1.05771 | 0.588686 | 0.633853 | 0.228046 | 0.539402 |
| 7 | 33.1928 | 33.397 | 0.288409 | 0.623673 | 0.397897 | 0.37538 | 0.461748 | 1.05771 | 1.41516 | 0.516718 | 0.144204 | 0.311836 |
| 8 | 34.3548 | 34.355 | 0.121321 | 0.312053 | 0.167657 | 0.187812 | 0.461384 | 1.05775 | 1.68094 | 0.819856 | 0.0606605 | 0.156026 |
| 9 | 35.735 | 35.875 | 0.0283244 | 0.159325 | 0.0391447 | 0.0958949 | 0.461641 | 1.05772 | 2.18967 | 2.37694 | 0.0141622 | 0.0796623 |
| 10 | 40.1396 | 40.281 | 0.126701 | 0.189177 | 0.174364 | 0.113863 | 0.46256 | 1.05771 | 2.68604 | 1.15E+00 | 0.0633503 | 0.0945883 |
| 11 | 42.3496 | 42.414 | 0.0365726 | 5.87E-02 | 0.0503749 | 0.0353152 | 0.462569 | 1.05777 | 2.17442 | 9.30E-01 | 0.0182863 | 2.93E-02 |
| 12 | 44.1377 | 44.138 | 0.0336649 | 0.0806076 | 0.0464097 | 0.0485165 | 0.462057 | 1.05771 | 1.75516 | 0.778532 | 0.0168325 | 4.03E-02 |
| 13 | 45.58 | 45.581 | 0.0261013 | 0.0640023 | 0.0360314 | 0.0385217 | 0.461871 | 1.05772 | 1.60232 | 0.785406 | 0.0130507 | 0.0320012 |
| 14 | 46.8973 | 47.037 | 0.166839 | 0.262804 | 0.229836 | 0.158171 | 0.462415 | 1.05775 | 1.92206 | 0.702736 | 0.0834193 | 0.131402 |
| 15 | 48.4796 | 48.34 | 0.0805324 | 0.149378 | 0.11072 | 0.0899084 | 0.463276 | 1.05771 | 1.50802 | 0.668302 | 0.0402661 | 0.0746889 |
| 16 | 49.719 | 49.579 | 0.195947 | 0.318546 | 0.270617 | 0.191728 | 0.460877 | 1.06E+00 | 1.33673 | 0.633853 | 0.097973 | 0.159273 |
| 17 | 50.8166 | 50.754 | 0.0970521 | 0.176652 | 0.13374 | 0.106325 | 0.462326 | 1.05771 | 0.916896 | 0.930086 | 0.0485258 | 0.0883261 |
| 18 | 52.4003 | 52.478 | 0.0806156 | 0.206069 | 0.111116 | 0.124024 | 0.462223 | 1.05776 | 1.2597 | 0.40648 | 0.0403078 | 0.103035 |
| 19 | 53.4357 | 53.231 | 0.0922898 | 0.192106 | 0.127283 | 0.115625 | 0.461915 | 1.05772 | 0.915971 | 1.60E+00 | 0.0461449 | 0.096053 |
| 20 | 56.1936 | 56.194 | 0.0394203 | 0.120501 | 0.0543974 | 0.0725278 | 0.461585 | 1.05771 | 1.41468 | 5.58E-01 | 0.0197101 | 0.0602505 |
| 21 | 57.357 | 57.229 | 0.0312772 | 0.097779 | 0.0429979 | 0.0588518 | 0.463085 | 1.05771 | 1.42569 | 1.77753 | 0.0156386 | 0.0488895 |
| 22 | 60.2567 | 60.524 | 0.0306937 | 0.124245 | 0.042281 | 0.0747804 | 0.462556 | 1.0600 | 1.92311 | 7.10E-01 | 0.0153469 | 0.0621226 |
| 23 | 61.8396 | 61.839 | 0.0504677 | 0.127182 | 0.0693947 | 0.0765486 | 0.463017 | 1.05771 | 1.67415 | 8.13E-01 | 0.0252339 | 0.0635908 |
| 24 | 63.2188 | 63.346 | 0.0602663 | 0.137834 | 0.0828782 | 0.0829596 | 0.462992 | 1.05772 | 1.24361 | 0.372037 | 0.0301332 | 0.0689171 |
| 25 | 64.2409 | 64.036 | 0.0869556 | 0.189557 | 0.120211 | 0.114089 | 0.459892 | 1.05774 | 1.35115 | 7.85E-01 | 0.0434778 | 0.0947785 |
| 26 | 65.3508 | 65.492 | 0.0561969 | 0.123173 | 0.0773253 | 0.0741362 | 0.462817 | 1.05771 | 1.66491 | 1.78E+00 | 0.0280985 | 0.0615866 |
| 27 | 70.0177 | 68.787 | 0.017741 | 0.0477106 | 0.0243679 | 0.0287161 | 0.46398 | 1.05772 | 0.106725 | 0.633851 | 0.00887049 | 0.0238553 |
| 28 | 71.9536 | 69.962 | 0.0277489 | 0.0496909 | 0.0381437 | 0.0299082 | 0.462659 | 1.05771 | 0.0110895 | 1.11612 | 0.0138745 | 0.0248454 |
| 29 | 72.5793 | 72.031 | 0.0181771 | 0.0581726 | 0.0250233 | 0.0350121 | 0.463178 | 1.05774 | 0.0219059 | 1.22633 | 0.00908853 | 0.0290863 |
| 30 | 74.2268 | 74.304 | 0.0294655 | 0.0674524 | 0.0404634 | 0.0405975 | 0.462069 | 1.05774 | 0.0450869 | 0.702741 | 0.0147328 | 0.0337262 |
| 31 | 75.8811 | 75.607 | 0.0352437 | 0.0688902 | 0.0484173 | 0.0414641 | 0.464209 | 1.05771 | 1.67346 | 0.89565 | 0.0176508 | 0.0344451 |
| 32 | 77.2671 | 77.267 | 0.0515207 | 0.114448 | 0.084582 | 0.0688826 | 0.379841 | 1.05774 | 1.50468 | 0.66827 | 0.0257604 | 0.0572239 |
| 33 | 78.5048 | 78.506 | 0.0347387 | 0.0794345 | 0.0479184 | 0.0478073 | 0.462148 | 1.05778 | -- | -- | 0.0173694 | 0.0397172 |
