# Supplementary material for: Cell behavior on silica-hydroxyapatite coaxial composite
Source: PLoS One. 2021 May 11;16(5):e0246256. doi: 10.1371/journal.pone.0246256 (PMC8112647; doi:10.1371/journal.pone.0246256)
Supplement: Attachment — Submitted filename: Response comments agosto.docx [file pone.0246256.s003.docx]

**Response comments**

**Reviewer 1**

Some typing errors and grammatical errors present throughout the manuscript. Methodology for in vivo study does not clearly stated the sample size. Objectives not clearly stated both in the abstract and introduction, especially not mention anything about characterization of the material and in vivo study.

Response: The corrections were made in the manuscript

**Reviewer 2**

In this research, investigate the cytotoxic behavior of electrospun SiO2-HA non-woven ceramic membranes. However, some issues regarding present study should be addressed before publication in PLOS ONE. It is recommended that the following be done to complete the article:

1-“Abstract” should be rewritten again by considering better grammar.

Response: The corrections were made in the manuscript

2- Generally, using chemical formulas such as ‘SiO2” is not usual in “Keywords”. Therefore, another keyword should be replaced instead of “SiO2”.

Response: The corrections were made in the manuscript

3- What does the surface area of SiO2-HA fiber indicate? Please discuss the issue?

Response: The corrections were made in the manuscript

4- Results of ATR-FTIR should be mentioned with references. Please examine the issue.

Response: The corrections were made in the manuscript

5- Statistical analysis or p-value is required for in vitro and in vivo tests. Please mention the issue in the manuscript.

Response: The corrections were made in the manuscript

6- Results of in vitro and in vivo should be expressed in “Conclusion”. “Conclusion” should be rewritten again.

Response: The corrections were made in the manuscript

7- To conclude, writing related to manuscript is weak. Please examine and use better grammar for it.

Response: The corrections were made in the manuscript

**Reviewer & Editor Comments**

Q1: In the abstract, you need to focus more on the quantitative information, not qualitative ones.

Response: The corrections were made in the manuscript

Q2: In the “Introduction” section, the authors were supposed to clear the answers of the following questions;

a. Which exact problem was supposed to be solved by the present research?

b. Which new achievement(s) was supposed to be obtained by the present research in comparison with the previous reports?

Response: The corrections were made in the manuscript

Q3: Title and subtitle of each section require numbering.

Response: The corrections were made in the manuscript

Q4:  Please describe the underlying reason behind selection of silica precursor at 10 w/v% and HA sol at 20 w/v% for the composite fibers.

Response: The corrections were made in the manuscript

Q5: You stated that " The surface area of the sintered SiO2-HA fibers was 5.77 m2/g, with an average pore width of 135.87 Å". How did you measure surface area and averages pore width of the sintered SiO2-HA fibers.

Response: The corrections were made in the manuscript

Q6: In the method section, how many times have you repeated the cell culture tests? Have you considered the repeatability of the tests?

Response: The corrections were made in the manuscript

Q7: Histogram analysis of SiO2-HA fiber composite to further confirm the average diameter of fiber composite and its distribution.

Response: The corrections were made in the manuscript

Q8: What do you mean by "vibrational modes assigned to "...725 cm-1 for P2O74-". There is no such band "P2O74-" in aforesaid wavenumber.

Response: The corrections were made in the manuscript

Q9: "vibrational modes of Si-O-Si" should be indicated in the ATR-FTIR analysis". In addition, your statement is not clear "according with IR and XRD the combination of SiO2 an HA does not introduce a differential or partial phase transformation of HA to another tricalcium phosphate".

Response: The corrections were made in the manuscript

Q10: I recommended the author to present the value of "crystallinity percentage", and "crystalite size" of HA and SiO2-HA in the table instead of Fig. 5.

Response: The corrections were made in the manuscript

Q11: the author should check the value of cell viability of fibroblasts incubated onto thermal-treated SiO2, HA and SiO2-HA. You present high cell viability of fibroblasts regarding SiO2, HA and SiO2-HA, these values should be compared with other study and literature. It is worth noting that you stated that  "SiO2 has a negative effect on certain cell cultures such as endothelial and fibroblast [29, 30]" which has a contradiction with  presented value in the graph (Fig. 6).

Response: The corrections were made in the manuscript

Q12: Y axis of Fig. 6 is missing. In addition, "*" sign is missing too inside the graph.

Response: The corrections were made in the manuscript

Q13: Table 1 and Table 2 regarding "Regression Analysis for Mixture Process Variable Model of values at 24 h and 48 h" could be presented in the supporting information.

Response: The corrections were made in the manuscript

Q14: Fig. 7 and Fig. 8 regarding maximum optimal response should merge together and be presented as Fig. 7a, b.

Response: The corrections were made in the manuscript

Q15: For benefit of readers, the schematic illustration regarding schematic demonstration of the interactions between the fibroblasts cell and surface of SiO2-HA fiber composite could be presented.

Q16: In conclusion part, you need to present more quantitative data in order to make it easier and convenient for readers to compare the specimens.

Response: The corrections were made in the manuscript

Q17: Moreover, for possible final paper acceptance, it is requested that authors improve the English

presentation, both grammar and style. Authors are strongly advised to have their paper revised for language by a native English or equivalent expert.

Response: The corrections were made in the manuscript

Q18: Surprisingly small reference to PLOS ONE in the literature despite the large relevant literature there. This should be improved. There are several important papers in the recent literature

" between 400-4000 cm-1" should change to " between 400-4000 cm-1"

" cells. [27,28]" should change to " cells [27,28]."

"membrane. [28]" should change to "membrane [28]."

Response: The corrections were made in the manuscript
