# Supplementary material for: Cell behavior on silica-hydroxyapatite coaxial composite
Source: PLoS One. 2021 May 11;16(5):e0246256. doi: 10.1371/journal.pone.0246256 (PMC8112647; doi:10.1371/journal.pone.0246256)
Supplement: Attachment — Submitted filename: Specific questions and comments response.docx [file pone.0246256.s005.docx]

Response for specific questions and comments are listed as follow:

Q1: As mentioned previously, you stated that "The surface area of the sintered SiO2-HA fibers was 5.77 m2/g, with an average pore width of 135.87 Å". How did you measure the surface area and averages pore width of the sintered SiO2-HA fibers? The procedure should be presented in the materials and methods.

Response: The corrections was made in the manuscript, In the revised manuscript, the text was left in red with the mention of the technique used.

Q2: As mentioned previously, the histogram analysis of SiO2-HA fiber composite to further confirm the average diameter of fiber composite and its distribution. The histogram analysis of SiO2-HA fiber composite was not provided.

Response: The corrections was made in the manuscript, In the revised manuscript, histogram analysis of SiO2-HA fiber composite was provided

Q3: You stated that "vibrational modes assigned to "...725 cm-1 for P2O74-"relate to the HA fibers spectrum. However, this vibration mode "P2O74-" is related to FTIR spectra of commercial product (Fluka β-TCP) according to the Ref [23].

Response: The corrections was made in the manuscript, In the revised manuscript, the text in red have a new information.

Q4: As mentioned previously, I recommended the author to present the value of "crystallinity percentage", and "crystallite size" of HA and SiO2-HA in the table instead of Fig. 5. However, the crystallite size of HA and SiO2-HA was not presented.

Response: The corrections was made in the manuscript, In the revised manuscript, the text in red have a new information.

Q5: "PO43- ions by HPO42-, Ca2+ by K+ or Mg2+, and OH- by F-, Cl-, Br-". Upper-case and lower-case should be used to indicate ions.

Response: The corrections was made in the manuscript,

Q6: For readers' benefit, the schematic illustration regarding schematic demonstration of the interactions between the fibroblasts cell and surface of SiO2-HA could be presented.

Response: The corrections was made in the manuscript, an schematic demonstration of the interactions between the fibroblasts cell and surface of SiO2-HA was presented.
